# Characterization of Bituminous Materials and Their Resistance to Fatigue Cracking by Dissipated Energy in Cyclic Tests: EBADE Test

**DOI:** 10.3390/ma18071587

**Published:** 2025-04-01

**Authors:** Félix E. Pérez-Jiménez, Rodrigo Miró, Adriana H. Martínez, Teresa López-Montero, María del Mar Colás, María González-González, Javier Cabeza-Barahona

**Affiliations:** 1 Department of Civil and Environmental Engineering, Universitat Politècnica de Catalunya, 08034 Barcelona, Spain; edmundo.perez@upc.edu (F.E.P.-J.); r.miro@upc.edu (R.M.); teresa.lopez@upc.edu (T.L.-M.); 2 Moeve, 08046 Madrid, Spain; marimar.colas@moeveglobal.com (M.d.M.C.); maria.gonzalezg@moeveglobal.com (M.G.-G.); javier.cabeza@moeveglobal.com (J.C.-B.)

**Keywords:** bituminous materials, fatigue cracking, dissipated energy, EBADE test

## Abstract

This paper presents the application of the same type of cyclic strain sweep test, the EBADE test, in the characterization of bitumens and mixes. This test employs a consistent methodology and set of parameters—namely, breaking stress, tenacity and dissipated energy at failure—to evaluate fatigue failure resistance in both materials. These parameters are intrinsically interrelated, and based on this relationship, the EBADE diagram was developed to establish a quantitative scale for resistance, ductility and tenacity. This scale facilitates the characterization and comparative analysis of bitumens and bituminous mixtures. The EBADE test and its associated diagram are applied to various penetration-grade and modified bitumens, as well as to the mixtures manufactured from them. The results demonstrate that the differentiating characteristics and advantages ob-served in the bitumen characterization are consistently reflected in the corresponding mixtures. Furthermore, the EBADE test, conducted across a range of temperatures, enables the generation of characteristic curves for both bitumens and mixtures within the EBADE diagram, facilitating a comparative analysis of their respective responses. The findings of this study validate the suitability of using dissipated energy for the characterization of the fatigue resistance of both bitumens and mixtures.

## 1. Introduction

The mechanical performance of asphalt mixtures is determined by the combined characteristics of the bitumen and the mineral skeleton. Bitumen plays a critical role in defining the mechanical properties as well as its resistance to deterioration through disintegration and cracking. Initially, bitumen was considered as a viscous material, and its mechanical behaviour was characterized in terms of consistency and viscosity. The contribution of bitumen to the asphalt mix was predominantly related to resistance to plastic deformation (consistency at high temperatures, recorded by Ring and Ball test) and to the brittle behaviour at low temperatures (Fraass test). However, it is now acknowledged that fatigue cracking of bituminous mixtures is a key performance factor during the pavement’s service life. Consequently, extensive research was conducted to evaluate the binder’s contribution to asphalt mixture performance, exploring the usefulness of parameters other than viscosity and consistency [1].

In the 1980s, Superpave specifications [2] began considering the whole mechanical behaviour of bitumen by including cyclic loading tests. Complex modulus and phase angle determined by DSR (Dynamic Shear Rheometer) in the Strategic Highway Research Programme (SHRP) developed in the USA [3] led to calculation of |G*|/sin δ parameter related to the resistance of the bitumen to plastic deformation and to the definition of the upper limit of service temperature. The ductility and modulus of the bitumen at low temperatures were also considered and assessed using the ductility tester and the BBR (Bending Beam Rheometer) flexural bending test, with the aim of determining the temperature at which the material behaviour changes from ductile to brittle. Fatigue cracking resistance was assessed by the elastic modulus (G″ = |G*|·sin δ) at 20 °C (or at the average service temperature). It was observed that mixtures with a stiff response and high elastic modulus, showed fatigue cracking problems. Recently, other rheological parameters that are more consistent with the performance of the mixtures are being used, such as the Glover-Rowe parameter (GRP = |G*| (cos δ)^2^/sin δ) or the Viscous to Elastic Transition Temperature (VETT, temperature at which G′ = G″) [4]. Although these parameters are related with the fatigue phenomena, they do not measure the actual cracking resistance of the bitumen nor that of the mixture.

The resistance to fatigue cracking of bituminous binders has also been studied by classical fatigue tests on bitumen using the DSR equipment [5]. Two procedures have been extensively explored: time sweep (TS test) and linear amplitude sweep (LAS) [6]. In TS, a bitumen specimen is subjected to a constant strain until its modulus decreases by half. It enables obtaining the fatigue law by testing a specimen at different strain levels. In LAS test, strain is continuously raised until failure and the dissipated energy is determined in each cycle [7,8,9]. By applying the VECD model, the fatigue law may be determined using LAS procedure [10,11]. These tests enable a better understanding of fatigue behaviour, but some limitations have been reported. In case of TS test, thixotropy, friction heating and edge flow constriction phenomena hinder the accurate determination of the initial modulus [12,13]. In the case of LAS procedure, some authors question the application of the LVECD theory to determine the fatigue law from the test results. The fatigue laws obtained by this procedure strongly differ from those obtained using the TS method, which is harsher [14].

In recent years, models have been proposed to determine the fatigue law of mixtures from the failure strain of bitumen. The GFTAB [4]. model states that the fatigue life of a bituminous mixture is proportional to the ratio of the failure strain of the bitumen used, FSC, to the strain of the bitumen in the mixture (FSC/ε), raised to an exponent related to the bitumen phase angle.

(1) $$N_{f}={\left(\frac{FSC}{\varepsilon }\right)}^{k_{1}(90/\delta )}$$

The value of the failure strain (FSC) of the bitumen is referenced by means of a performance ratio factor FFPR to the failure strain of a typical bitumen (FSC*) taken as a reference. The effective design strain of the bitumen is obtained from the strain of the mix and the percentage of the mix volume filled by the binder.

(2) $$N_{f}={\left(\frac{FFPR\cdot {FSC}^{*}}{\varepsilon }\right)}^{k_{1}(90/\delta )}$$

where:

*Nf*: number of cycles to failure;

*FFPR*: ratio of the strain capacity of a given binder to the average or typical strain capacity;

*FSC*: average or typical fatigue strain capacity (%), analogous to failure strain, =4.45 × 10^6^ |G*|^−0.806^;

*ε*: effective strain in binder (%) = mix strain/(effective binder content by volume, or V_be_/100);

*k*_1_: constant determined through statistical analysis to be 2;

*δ*: binder phase angle (degrees).

The FFPR value can be determined by testing the bitumen with the SDENT test:

(3) $$FPR=\frac{Extension}{{17.4\cdot S}^{-0.334}}$$

*Extension*: extension of the specimen (mm);

*S*: initial stiffness of the specimen in Newtons per meter (N/m).

The Road Research Laboratory (Universitat Politécnica de Catalunya) developed a tension-compression cyclic strain sweep test named EBADE test (where EBADE stands for Ensayo BArrido de DEformaciones in Spanish), focused on determining the fatigue failure strain of both bitumen and mixes. EBADE test enables assessing and comparing the fatigue resistance of different bituminous materials and can be performed at different temperatures. Fatigue laws for mixtures obtained by this test are very similar to those obtained by conventional fatigue tests. EBADE does not use the GFTAB model and the deterioration is observed at each strain level [15]. EBADE test also provides the energy dissipated by the material until failure and these two parameters are related—as shown in this paper—to the maximum stress offered by the material during its breaking process. This relationship was observed in the analysis of the results obtained in the Road Research Laboratory in the application of the EBADE test to the characterization of bitumen and mixes and was subsequently confirmed when applied in a more comprehensive study carried out in collaboration with Moeve.

The interrelationship between fatigue failure strain, maximum stress and dissipated energy density allows for the construction of the EBADE diagram, which graphically illustrates the temperature-dependent variation of these three properties through characteristic curves. This graphical representation facilitates a comprehensive assessment of both bitumen and bituminous mixtures, enabling a comparative analysis of their behavior and mechanical response. These diagrams demonstrate that, consistent with findings reported in NCHRP 2022 [4], as temperature decreases, the modulus increases and the failure strain decreases. However, the dissipated energy exhibits a maximum, which indicates the optimal temperature or temperature range for the effective utilization of the bitumen or mixture.

The objectives of this work were to validate and obtain the relationship in the EBADE fatigue test between the dissipated energy density, the failure strain and the maximum stress, to define and implement a diagram that allows a clear and appropriate way to characterize bitumens and mixes, and to show the advantages of this procedure.

In this work, the EBADE test and diagram are applied to the characterization of different types of penetration and modified bitumen, as well as to the BBTM mixtures manufactured with them. The differentiating characteristics and advantages observed in the bitumen characterization are consistently reflected in the corresponding BBTM mixtures. By conducting the EBADE test across a range of temperatures, characteristic curves for both the bitumens and mixtures are generated within the EBADE diagram, facilitating a comparative analysis of their respective mechanical responses.

## 2. Methodology

The methodology used in the fatigue analysis and characterization of bitumens and bituminous mixes is based on the EBADE test and diagram. Firstly, for a better understanding of the methodology, the EBADE test and the relationship obtained between the dissipated energy density, the failure strain and the maximum stress from the tests carried out at the Road Research Laboratory of the Universitat Politècnica de Catalunya (Barcelona, Spain) are presented. Then, there is a stage with the validation and better definition of this relationship with the tests carried out at the Moeve Laboratory (Madrid, Spain). Finally, the diagram implemented and its application to the characterization and assessment of the materials tested are described.

The EBADE test is a tension-compression cyclic strain sweep test, applied to bitumen, mastics and bituminous mixes, for analyzing its behaviour under repeated cyclic loads [16]. A stepwise strain sweep is carried out by increasing the strain level every 5000 cycles. The test can be carried out at different temperatures with the aim of observing its effect on the response of the bituminous materials.

**Figure 1:**
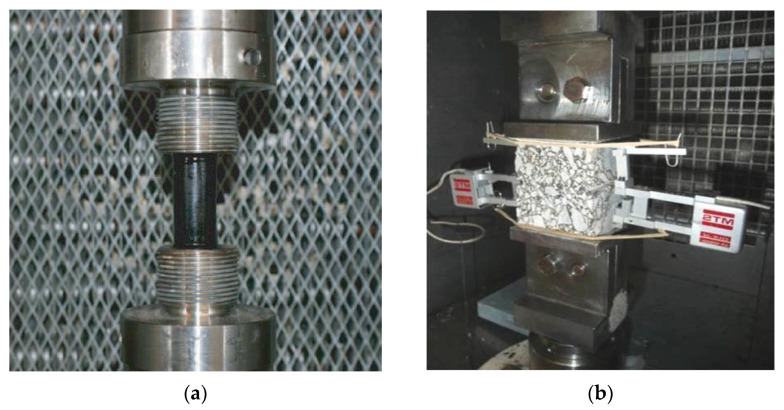
Applied EBADE test: (**a**) cylindrical bitumen specimens; (**b**) prismatic specimens of mixes [15].

In the case of bitumen and mastics, Figure 1a, the tests are performed on cylindrical specimens, manufactured by pouring the hot product into cylindrical molds of 20 mm diameter and 40 mm high. The initial strain is 0.00076 (mm/mm) and the strain is increased equally every 5000 cycles.

For testing asphalt mixes, the test is carried out using a prismatic specimen with 6 cm height and 5 × 5 cm^2^ base (Figure 1b), obtained by sawing cylindrical or prismatic specimens, compacted either in Marshall or wheel tracker type molds. In this case, the displacement level applied to the specimen is ±25 microns (µm) at the start, which is increased by the same magnitude every 5000 cycles.

The test can be carried out at any temperature, although the range normally used was between −5 and 10 °C. The tests were carried out at a frequency of 10 Hz on bitumen, mastics, and mixes.

How the applied stress varies is recorded in the test. From this record, the variation of the maximum stress applied in each cycle and associated modulus are obtained. This test also determines, from the hysteresis loop area, the dissipated energy in each cycle.

**Figure 2:**
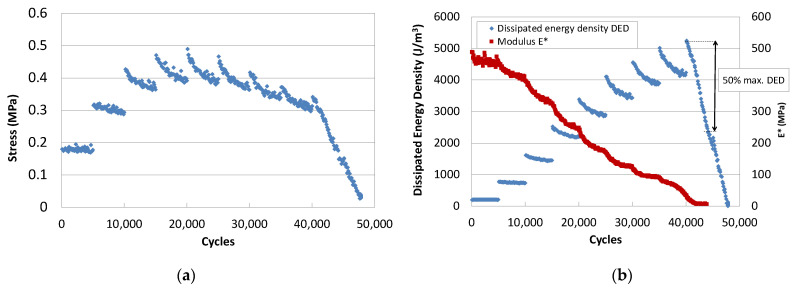
EBADE test results: (**a**) stress vs. number of cycles; (**b**) modulus and dissipated energy versus number of cycles [15].

The evolution of the measured parameters obtained in this test is plotted in Figure 2a,b. The stress, Figure 2a, sharply increases each time the strain level increases, until a step is reached where a maximum occurs. It describes the typical breakage curve of a tensile test. The stress decreases within each strain step.

It can be observed that the modulus continuously decreases and this behaviour is more pronounced in the first cycles of the initial steps, Figure 2b. Afterwards, it descends continuously and more slowly, until a sudden drop occurs at the end of the test due to failure.

The dissipated energy decreases within each step and increases with the strain level, until a limit strain level is reached and dissipated energy starts to drop rapidly. The failure strain is associated with the load step at which the dissipated energy drops below 50% of the maximum.

**Figure 3:**
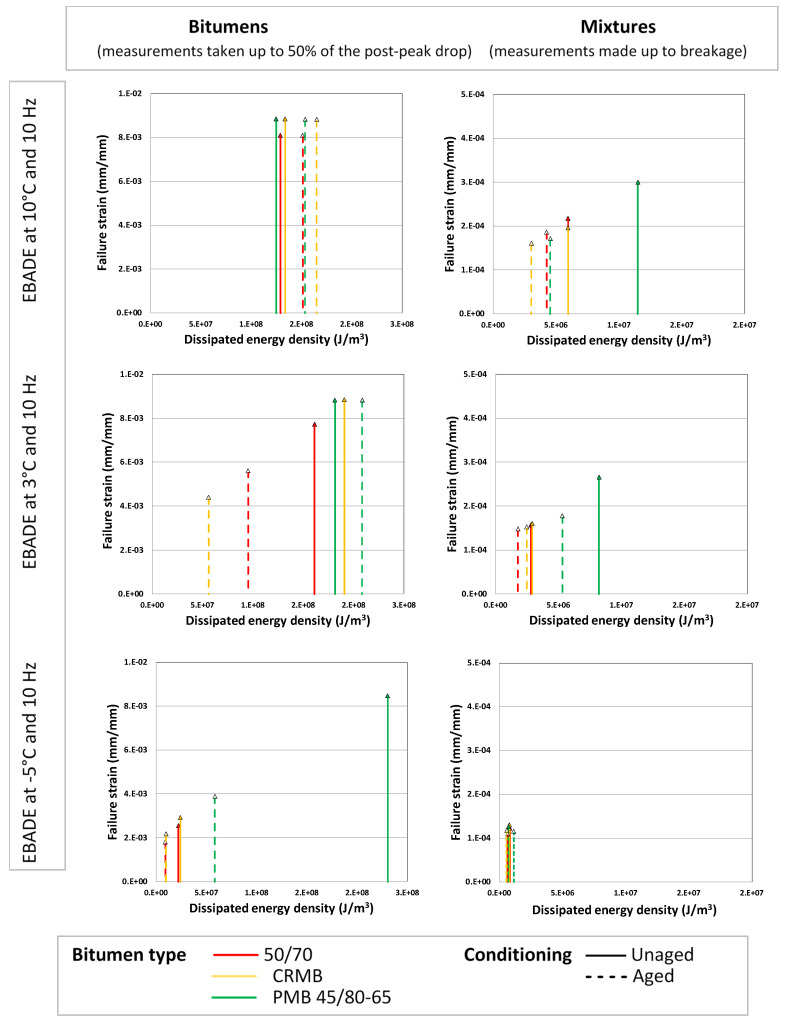
Failure strain, FS, versus the dissipated energy density up to failure, DED.

The EBADE test was used since 2010 in the characterization of bitumen and mixtures at the Road Research Laboratory of the UPC [17]. This test was mainly aimed at obtaining the failure strain of bitumen and mixtures, FS and the total Dissipated Energy Density, DED, obtained as the sum of the energies dissipated in each cycle until failure. Figure 3 shows results corresponding to the test of three types of 50/70 bitumen with different nature but similar penetration at 25 °C: refinery conventional bitumen, crumb rubber modified bitumen and polymer modified bitumen (PMB). The failure strains were obtained for each bitumen at three temperatures (10, 3 and −5 °C) and for two ageing conditions.

AC type mixtures (asphalt concrete) were manufactured with the three types of bitumen and using the same bitumen content in all cases (4.5% of the aggregate weight). The mixtures were also tested at three temperatures and two ageing states: unaged and subjected to an ageing process prior to their manufacture of 24 h in the oven. These results are also shown in Figure 3.

The analysis of the results obtained after testing bitumen clearly shows the effect of temperature and ageing on their response. The lower the temperature and the more aged the bitumen, the lower the failure strain. The type of bitumen also affects the failure strain, being higher for polymer modified PMB 45/80-65 bitumen. This difference cannot be observed for some bitumen at 3 and 10 °C because the maximum strain applied in the test was limited.

The dissipated energy is also temperature dependent, showing a maximum value at intermediate temperatures for unaged bitumen 50/70 (conventional) and BC (crumb rubber modified).

The response of the mixtures replicates the one observed in the bitumen, highlighting the effect of temperature, ageing and bitumen type on the behaviour.

Initially, in the analysis of results, only ductility (failure strain) and dissipated energy density were taken into account as the parameters that could best characterize the response to fatigue cracking failure. However, deeper analysis of the results shows that the parameters of ductility, maximum strength and dissipated energy are related. This enables a better analysis of the results and facilitates the comparison of the responses to fatigue failure of bitumen and asphalt mixtures.

## 3. Relationship Between Ductility, Maximum Strength and Dissipated Energy

A good correlation was obtained between the dissipated energy and the product of the maximum strength and the failure strain squared.

(4) $$DED={FS}^{2}\cdot MS$$

**Figure 4:**
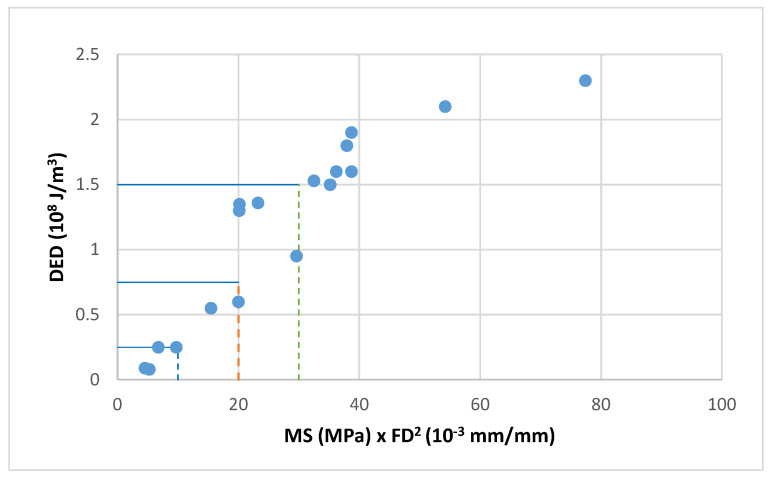
Dissipated energy versus the product of failure strain squared and maximum strength for bitumen 50/70, BC 50/70 and PMB 45/80-65.

**Figure 5:**
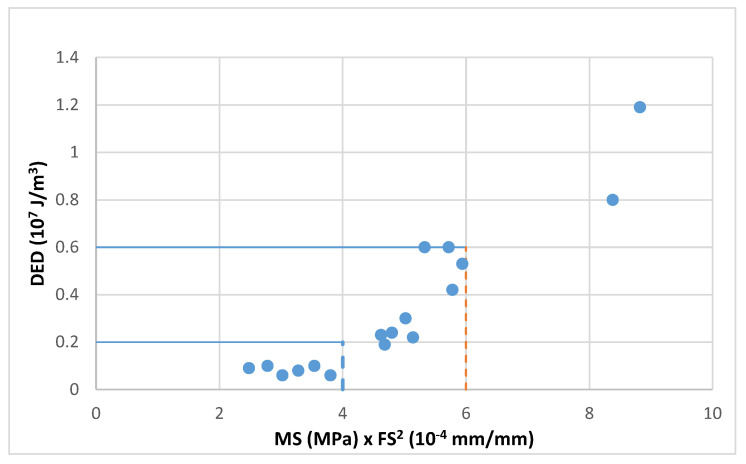
Dissipated energy versus the product of failure strain squared and maximum strength for AC mixtures manufactured with bitumen 50/70, BC 50/70 and PMB 45/80-65.

This correlation can be observed in Figure 4 and Figure 5 where these values have been plotted for the three tested bitumens and mixes (aged and unaged and at the three test temperatures −5, 3 and 10 °C). These graphs show, both in bitumens and mixes, the existing trend between the product MS·FS^2^ and the dissipated energy density DED.

The reference units have been changed in these figures to achieve simpler scales for the parameters used.

- For bitumens DED: 10^8^ J/m^3^, MS: MPa and FS: 10^−3^ mm/mm
- For mixtures DED: 10^7^ J/m^3^, MS: MPa and FS: 10^−4^ mm/mm

The energy dissipated by the bitumen exceeds that of the mixture by more than ten times and this difference is also seen with the failure strain.

## 4. Validation of the Study

EBADE test was applied to seven different bitumen grades produced by MOEVE: 15/25, 50/70, BC50/70, PMB 10/40-70, PMB 45/80-60 C, PMB 45/80-65 and PMB 45/80-75. Characterization is shown in Table 1. The test was applied to these seven bitumens, aged (RTFOT and PAV) and unaged, at three temperatures: 10, 5 and 0 °C, and the relationship between failure strain FS, maximum stress MS and dissipated energy density DED was analyzed.

BBTM mixtures were manufactured with 5.0% binder by weight of mix, with the following bitumen: B50/70, PMB 45/80-65 and PMB 45/80-75. The mixtures were also tested at three temperatures: 20, 10 and 0 °C.

Table 2 shows the gradation of the aggregates used (porphyry coarse aggregate, limestone fine aggregate and calcium carbonate filler). The specimens were manufactured with 5.0% bitumen by weight of mix and compacted with 17% voids.

### Relationship Between the Three Basic Parameters: Failure Strain, Maximum Stress and Dissipated Energy

These tests again show the relationship of the dissipated energy density DED with the maximum stress MS and the failure strain FS in both mixtures and bitumens.

(5) $$DED=K\cdot {MS\cdot FS}^{2}$$

**Figure 6:**
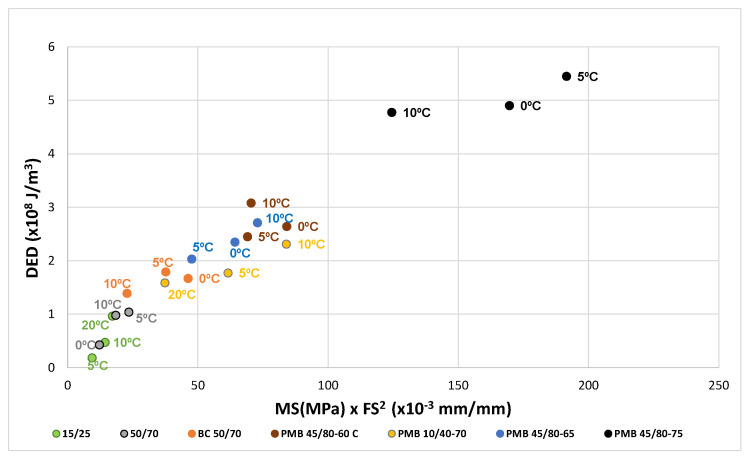
Dissipated energy density as a function of the product MS × FS^2^. Unaged bitumens.

The test results of the unaged bitumen are plotted in Figure 6. The dissipated energy density DED is plotted as 10^8^ J/m^3^ on the abscissa axis, and the product of the maximum stress MS (MPa) times the square of the failure strain FS (mm/mm) in thousandths (0.001) on the ordinate axis. It can be seen from this correlation that a factor K = 1/25 can be established which relates energies with the product of the failure strain squared by the maximum stress.

**Figure 7:**
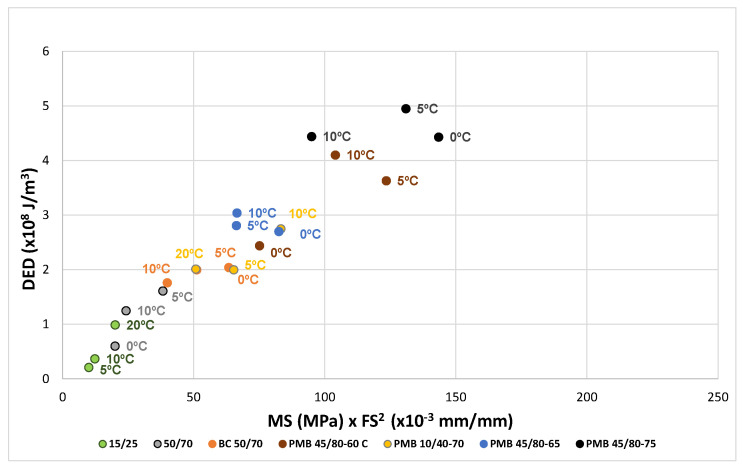
Dissipated energy density as a function of the product MS × FS^2^. RTFOT aged bitumens.

**Figure 8:**
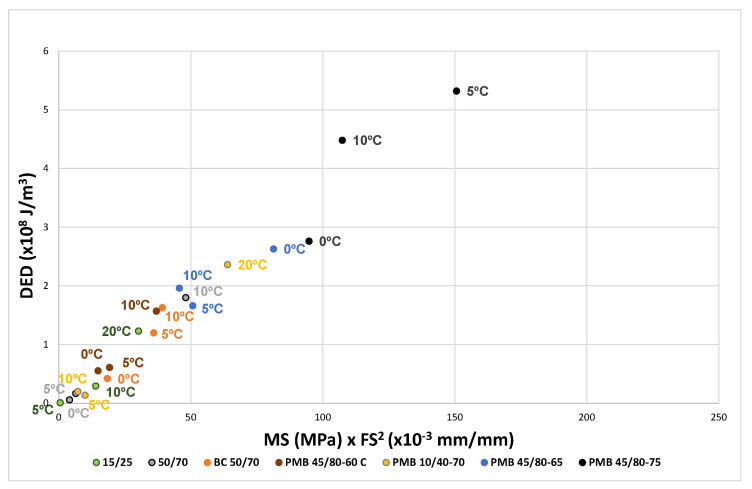
Dissipated energy density as a function of the product MS × FS^2^. RTFOT + PAV aged bitumens.

The same factor 1/25 corresponds to the test results of the aged bitumens, as can be seen in Figure 7 and Figure 8.

**Figure 9:**
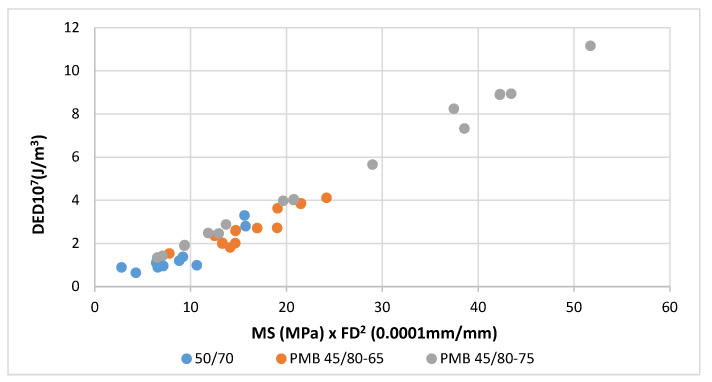
Dissipated energy density versus the product MS × FS^2^. BBTM mixtures with unaged bitumen 50/70, PMB 45/80-65 and PMB 45/80-75 (0, 10 and 20 °C).

The same applies to the mixtures tested, Figure 9. In this case, the results of three mixtures prepared with 50/70, PMB 45/80-65 and PMB 45/80-75 bitumens are shown. The relationship between the energy and the product of maximum stress and failure strain squared is also shown. Now the factor relating these parameters is 1/5.

This relationship allows the incorporation in the stress-strain graphs of iso-tenacity curves indicating the tenacity of the tested material, named EBADE diagram.

## 5. EBADE Diagram—Comparison of the Behaviour of Bitumen and Mixes—Characteristic Curves

In the comprehensive characterization of bituminous materials, it is of great importance to elucidate the temperature-dependent transition of their properties, specifically from a ductile and tenacious state to a rigid and brittle state as temperature decreases. Conversely, an elevation in temperature leads to a reduction in consistency and cohesive strength.

The application of the EBADE test enables the analysis of both bitumens and mixtures across a range of temperatures. The graphical representation of these results within the EBADE diagram yields a characteristic curve for each material tested, effectively illustrating their respective behaviors and facilitating a comparative analysis of their mechanical responses.

By defining MS × FS^2^ = constant, energy zones associated with these tests are defined, which allow assessing how the dissipated energy and tenacity of the materials change with temperature, the EBADE diagram.

The curves used for bitumens have the following values for MS·FS^2^: 5; 25; 50; 75 and 125.

These curves correspond to the following energy levels DED (10^8^ J/m^3^): 0.2; 1; 2; 3 and 5.

**Figure 10:**
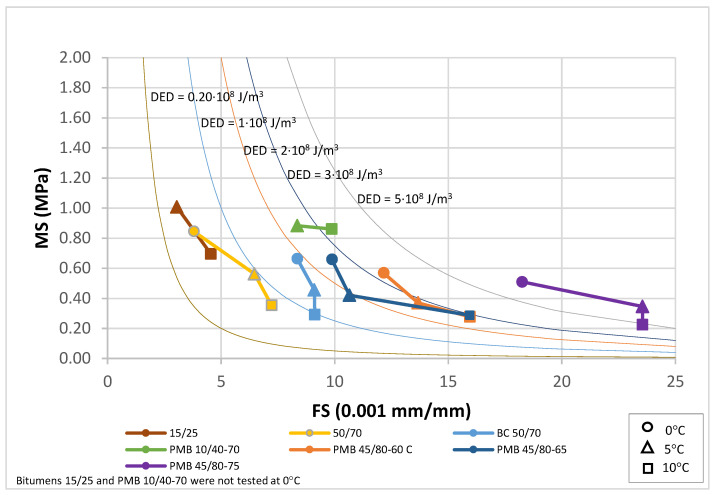
Characteristic curve. Unaged bitumens (0, 5 and 10 °C).

**Figure 11:**
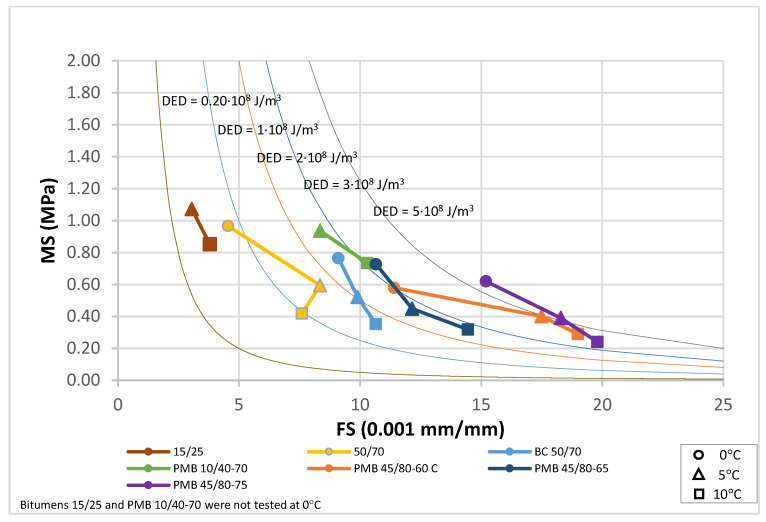
Characteristic curve. RTFOT aged bitumens (0, 5 and 10 °C).

**Figure 12:**
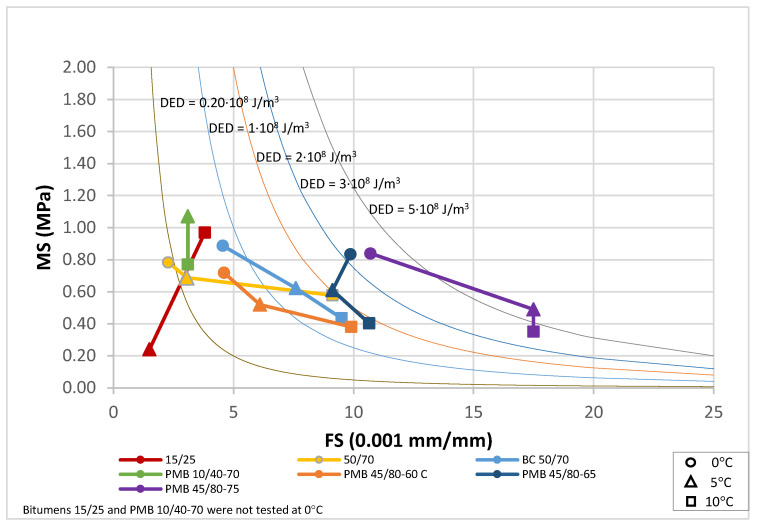
Characteristic curve. RTFOT +PAV aged bitumens (0, 5 and 10 °C).

The results for the bitumens before and after ageing have been plotted in Figure 10, Figure 11 and Figure 12. For each binder, its characteristic curve was plotted with the results of the tests carried out at 0, 5 and 10 °C. These diagrams allow assessing and comparing the behaviour of the tested bitumens in a simple and straightforward way.

The representation of the characteristic curves of the seven bitumens in the EBADE diagram, Figure 10, clearly shows the differences between conventional penetration graded bitumens and modified bitumens. It can be observed that modification with elastomers yields higher ductility and tenacity. The curves also show the effect of the hardness and penetration of the bitumens. Thus, the hard bitumen 15/25 and PMB 10/40-70 show at 5 and 10 °C a higher resistance than the rest of the bitumens at 0 °C.

The curves of conventional bitumens 15/25 and 50/70 show the differences in their response as temperature changes. At 10 and 5 °C the harder bitumen has a more brittle and less tenacious response.

The addition of crumb rubber to bitumen (BC 50/70) makes it more ductile, able to maintain the ductility at lower temperatures and have a higher tenacity than bitumen 50/70, which has a similar penetration.

Polymer modified bitumens show the best response. They are more ductile and tenacious bitumen. They also allow harder bitumen to be obtained, with greater strength and modulus, without losing ductility, like bitumen PMB 10/40-70, designed for high modulus mixtures. It is important to note the effect of the incorporation of polymers in increasing the dissipated energy, especially in the case of bitumen PMB 45/80-75.

The tenacity defined by the curves indicate that conventional bitumens are in the range 0.2–1.0 (10^8^ J/m^3^), crumb rubber bitumen BC 50/70 is in the range 1.0–2.0 (10^8^ J/m^3^), polymer modified bitumens PMB 10/40-70, PMB 45/80-60 C and PMB 45/80-65 are within 2.0–3.0 (10^8^ J/m^3^), and highly modified PMB 45/80-75 is greater than 5.0 (10^8^ J/m^3^).

The EBADE plot of the characteristic curves of bitumens aged after RTFOT and PAV, Figure 11 and Figure 12, shows the progressive loss of ductility and tenacity of all bitumens. This loss is more pronounced in the penetration bitumens than in the polymer modified bitumens. It can also be seen that the maximum stress of the bitumens tends to increase but does not exceed 1 MPa. In other words, ageing has a strong impact on ductility and increases strength to a lesser extent and up to a limit. This makes the bitumen increasingly brittle and less tenacious and decreases their resistance to cracking.

The fatigue behaviour of aged binders in terms of the tenacity determined by EBADE test at 10 °C shows the same trend as the parameter G″ = |G*|·sin δ, which is determined by DSR at the same temperature. Both procedures show the effect of penetration of the fresh binder in conventional bitumens, but G″ = |G*|·sin δ is not able to distinguish modified and conventional bitumens with the same penetration in an effective way while EBADE test reveals a better performance of PMBs and crumb rubber modified bitumens. Although conventional and modified bitumens with similar penetration may have similar modulus and phase angle in the early stages of the test, they show different evolution during the strain sweep.

**Figure 13:**
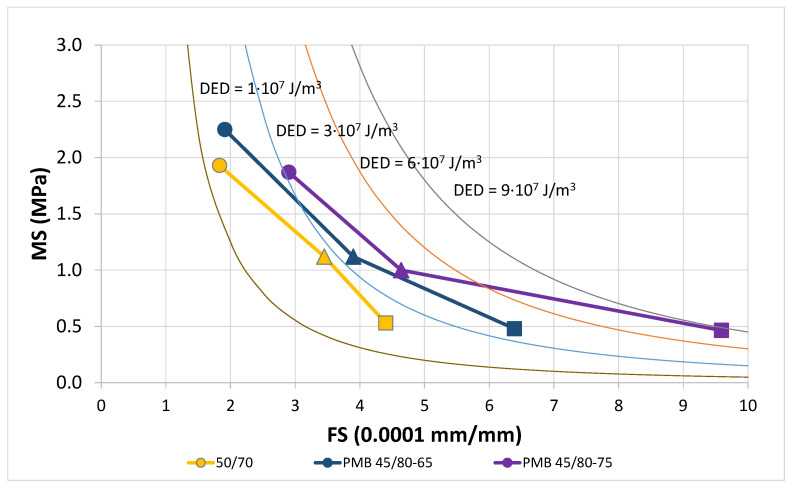
Characteristic curve. Mixture BBTM with bitumen 50/70, PMB 45/80-65 and PMB 45/80-75 (0, 10 and 20 °C).

The EBADE diagram implemented to represent the characteristic curves of BBTM mixtures, as shown in Figure 13, presents different scales and energy curves than those used for the bitumens in order to take into account the natural lower ductility of the mixtures, the lower dissipated energy and the different ratio between the maximum stress MS times the square of the failure strain FS and the dissipated energy density DED (K = 1/5).

The unit for the failure strain is 0.0001 mm/mm, ten times lower than that of bitumens and the curves used for the energy levels. The values of MS·FS^2^ are: 5; 15; 30 and 45.

These curves correspond to lower energy levels as well. The values of DED (10^7^ J/m^3^) are: 1; 3; 6 and 9.

The characteristic curves clearly demonstrate the difference in behaviour of the three analyzed mixtures, as well as the effect of the binder type used. The differentiation observed among the mixtures is analogous to that recorded among the binders.

The use of highly modified bitumen PMB 45/80-75 provides more ductile and tougher mixtures at all three test temperatures. The greatest difference is observed at 20 °C and decreases with decreasing temperature. The mix with modified bitumen PMB 45/80-65 also shows a better response than the one made with conventional bitumen 50/70. All three mixtures have a similar maximum stress at the three test temperatures.

The EBADE test allows obtaining directly the failure strain of bitumens and mixtures. By applying the GFTAB model methodology, the fatigue laws of the mixtures could be established from the strain obtained for the bitumens or directly from the failure strain of the mixtures.

Fatigue laws are of significant importance for the calculation of the fatigue life of pavement sections in which bituminous mixtures are employed. These laws facilitate the analysis of the influence of layer thicknesses on pavement life. However, they do not provide a direct assessment of the intrinsic fatigue resistance of the mixture, as the modulus must also be taken into account.

Consequently, the methodology employed herein proceeds directly to the application of the EBADE diagram. Given the inherent interrelation between tenacity, maximum strength (which correlates directly with modulus) and failure strain, the EBADE diagram facilitates a comparative analysis of these three critical parameters, while simultaneously elucidating their temperature-dependent evolution. Notably, tenacity, representing the energy dissipated prior to failure, serves as the most significant parameter for evaluating the fatigue cracking resistance of bitumens or bituminous mixtures. Specifically, an elevated tenacity value indicates enhanced resistance to fatigue failure, reflecting a greater energy requirement for material fracture.

Table 3 shows the DED values at 10 °C for both bitumens and mixtures manufactured with them, where the same order of quality observed in bitumens can be seen in mixtures.

## 6. Conclusions

The EBADE test is a cyclic controlled strain test that enables determining the maximum stress of the material (MS), the failure strain (FS) and the energy applied to and dissipated during the test (DED), which can be applied both to bitumen and mixtures.

Dissipated energy density (DED) is related with failure strain and maximum stress as DED = k·MS·FS^2^. It is a useful parameter to assess fatigue resistance of both bituminous binders and asphalt mixes, since it considers the contribution of the viscosity and ductility. The higher the value of the dissipated energy DED, the higher the resistance to fatigue cracking of the material.

The EBADE diagram plots stress (MS) and strain (FS) with the iso-tenacity curves and enables characterizing the mechanical behaviour (modulus, related to its maximum strength MS) and the resistance to fatigue cracking (ductility FS and tenacity DED). The representation of the EBADE test results at different temperatures in the EBADE diagram provides the characteristic curve of the material.

The EBADE test shows the effect of penetration of bitumens and temperature on the fatigue behaviour as well as the impact of modification with polymers and/or crumb rubber. PMBs show the best ductility and tenacity performance, while crumb rubber enhances these parameters compared to conventional bitumens in a lower extent.

This methodology consistently demonstrates a uniform order of evaluation and classification for both bitumens and the bituminous mixtures fabricated with them. For instance, at 10 °C, for bitumens 50/70, PMB 45/80-65 and PMB 45/80-75, the DEDs are 1.1 × 10^8^ J/m^3^, 3.0 × 10^8^ J/m^3^ and 4.8 × 10^8^ J/m^3^, respectively. Similarly, the DEDs for the corresponding mixtures exhibit an analogous trend to that observed in the bitumens, with values of 2.1 × 10^7^ J/m^3^, 2.6 × 10^7^ J/m^3^ and 3.9 × 10^7^ J/m^3^, respectively.

The application of this methodology facilitates the establishment of specifications aimed at enhancing the response of bitumens and mixtures to this deterioration mechanism. This study shows the efficacy of utilizing energy for characterizing the fatigue resistance of both bitumens and mixtures.

In contrast to conventional methods for assessing the fatigue resistance of bitumens, which often provide indicative results and may fail to detect the influence of modifiers, the EBADE method is based on the actual fatigue behavior of both bitumens and mixtures.

## Figures and Tables

**Table 1:** Characteristics of the bitumen used in this study.

Property	Standard	15/25	50/70	BC 50/70	PMB 10/40-70	PMB 45/80-60 C	PMB 45/80-65	PMB 45/80-75
Penetration (0.1 mm)	EN 1426	21	61	58	20	46	58	55
Softening point R&B (°C)	EN 1427	60.7	49.6	55.8	72.9	63	68	81
Cohesion (J/cm^2^)	EN 13589	-	-	-	4.4 *	0.16 ***	5.7 **	7.6 **
Fraass breaking point (°C)	EN 12593	−4.5	−10.5	−14	−6.1	−23	−15	−18
Elastic recovery at 25 °C (%)	EN 13398	-	-	47.5	77	62.9	89.7	90
Storage stability		-						
Δpenetration (dmm)	EN 13399	-	-	−3.8	−0.73	−6.2	−0.23	1.13
ΔR&B (°C)	EN 13399	-	-	−4.5	−0.36	−5	−0.22	0.21
Flash point (°C)	EN ISO 2592	332	320	283	319	250	300	299
Solubility (%)	EN 12592	99.8	99.9	-	-	-	-	-
Durability (RTFOT)	EN 12607-1							
Δmass (%)	EN 12607-1	0.18	0.24	0.4	0.22	0.62	0.3	0.27
Retained penetration (%)	EN 1426	70	58.4	65.8	75.5	73.2	66.2	75
Δ R&B (°C)	EN 1427	6.7	6.7	8.6	6.5	8.8	3.8	1.7
Fatigue cracking (PAV)	EN 14769							
G*.sen δ at 20 °C (Pa)	EN 14770	1.44 × 10^7^	4.37 × 10^6^	2.96 × 10^6^	1.22 × 10^7^	3.28 × 10^6^	3.28 × 10^6^	3.06 × 10^6^

* at 15 °C, ** at 5 °C,*** at 25 °C.

**Table 2:** Gradation of aggregates used in BBTM mixtures.

Sieve (mm)	16	11.2	8	4	2	0.5	0.063
Passing (%)	100	93	65	20	18	10	4.5

**Table 3:** Values of DED for bitumens and mixtures at 10 °C.

	50/70	PMB 45/80-65	PMB45/80-75
DED Bitumens (10^8^ J/m^3^)	1.1	3.0	4.8
DED Mixtures (10^7^ J/m^3^)	2.1	2.6	3.9

## Data Availability

The datasets presented in this article are not readily available because they are property of the authors.
